# The role of m^6^A modification during macrophage metabolic reprogramming in human diseases and animal models

**DOI:** 10.3389/fimmu.2025.1521196

**Published:** 2025-02-18

**Authors:** Huiling Wang, Peiqi Xu, Kai Yin, Shengjun Wang

**Affiliations:** ^1^ Department of Laboratory Medicine, Jiangsu Province Engineering Research Center for Precise Diagnosis and Treatment of Inflammatory Diseases, The Affiliated Hospital of Jiangsu University, Zhenjiang, China; ^2^ Department of Immunology, Jiangsu Key Laboratory of Laboratory Medicine, School of Medicine, Jiangsu University, Zhenjiang, China; ^3^ Department of General Surgery, Affiliated Hospital of Jiangsu University, Institute of Digestive Diseases, Jiangsu University, Zhenjiang, China

**Keywords:** M1/M2 macrophage reprogramming, glycolysis, tricarboxylic acid cycle, oxidative phosphorylation, fatty acid oxidation, cholesterol transport

## Abstract

Macrophage metabolic reprogramming refers to the process by which macrophages adjust their physiological pathways to meet survival and functional demands in different immune microenvironments. This involves a range of metabolic pathways, including glycolysis, the tricarboxylic acid cycle, oxidative phosphorylation, fatty acid oxidation, and cholesterol transport. By modulating the expression and activity of key enzymes and molecules within these pathways, macrophages can make the transition between pro- and anti-inflammatory phenotypes, thereby linking metabolic reprogramming to inflammatory responses and the progression of several diseases, such as atherosclerosis, inflammatory bowel disease (IBD), and acute lung injury (ALI). N6-methyladenosine (m^6^A) modification has emerged as a critical regulatory mechanism during macrophage metabolic reprogramming, broadly affecting RNA stability, translation, and degradation. Therapeutic strategies targeting m^6^A modification can regulate the onset of metabolic diseases by influencing macrophage metabolic changes, for instance, small molecule inhibitors of methyltransferase-like 3 (METTL3) can affect glucose metabolism and inhibit IBD. This review systematically explores recent findings on the role and molecular mechanisms of m^6^A modification during macrophage metabolic reprogramming in human diseases and animal models, underscoring its potential as a therapeutic target for metabolic diseases.

## 1 Background

Macrophages are a type of immune cell widely distributed across various organisms, playing a central role in immune response and homeostasis. They are found in a variety of species, ranging from invertebrates to vertebrates, and are involved in processes such as pathogen defense, tissue repair, and immune regulation. However, in mammals, including both mice and humans, macrophages exhibit a higher level of functional complexity, with their polarization states and metabolic reprogramming being particularly important in diseases such as inflammation, metabolic disorders, and cancer.

**Figure 1:**
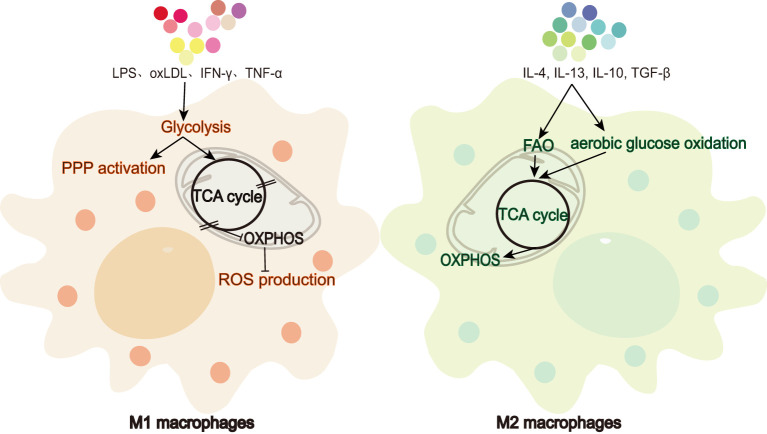
Metabolic differences between M1 and M2 macrophages. M1 macrophages favor glycolysis and ROS production while limiting TCA cycle and OXPHOS. In contrast, M2 macrophages rely on FAO, aerobic glucose oxidation, TCA cycle, and OXPHOS.

Macrophages, as one of the primary responders of the immune system in mammals, are key participants in innate immunity and serve as a bridge between innate and adaptive immune response through the antigen presentation process (1). These cells exhibit notable plasticity, with non-polarized M0 macrophages polarizing into pro-inflammatory M1 or anti-inflammatory M2 cells depending on the environmental cues, allowing them to play pivotal roles in inflammation, tissue repair, and disease progression (2). All cells require sufficient and appropriate nutrients and oxygen to maintain metabolic homeostasis. Macrophage metabolic reprogramming is an activity carried out by optimizing the physiological pathways of macrophages in response to their different metabolic demands during polarization. After polarization to the M1 or M2 phenotype, macrophages show modulated metabolic efficiency, reflected in different pathways and metabolite levels, as illustrated in **Figure 1**. Upon stimulation by factors such as lipopolysaccharide (LPS), interferon gamma (IFN-γ), tumor necrosis factor alpha (TNF-α), and oxidized low-density lipoprotein (oxLDL), macrophages are activated into the M1 phenotype and play a role in pro-inflammatory and antimicrobial responses (3). Glycolysis is the primary metabolic pathway used by M1 macrophages (4). Upon activation, these cells exhibit a Warburg-like effect (5) to meet their energy demands, shifting from reliance on oxidative phosphorylation (OXPHOS) to a more glycolytic-dependent pathway. The dependence of M1 cells on glycolysis for ATP production is due to blocks in the tricarboxylic acid cycle (TCA cycle), which limit the production of NADH and FADH2 required for the electron transport chain, thereby inhibiting OXPHOS (6). In contrast, upon stimulation by cytokines such as interleukin-4 (IL-4), interleukin-13 (IL-13), interleukin-10 (IL-10), and transforming growth factor-beta (TGF-β), macrophages are polarized into the M2 phenotype, which can be further subdivided into M2a, M2b, M2c, and M2d subtypes, contributing to anti-inflammatory responses, tissue repair, and parasitic infections (7, 8). Aerobic glucose oxidation and fatty acid oxidation (FAO) are the primary metabolic pathways used by M2 macrophages. Oxidative metabolism is also significantly enhanced in M2 cells (9, 10). Unlike the demands of energy metabolism in M1 macrophages, M2 cells maintain an intact TCA cycle, thus guaranteeing a smooth process of ATP production by OXPHOS (11, 12). Indeed the metabolic process in macrophages involves more than just changes in metabolite levels and ATP production; it also influences macrophage phenotype through the regulation of transcriptional and post-transcriptional events.

N6-methyladenosine (m^6^A) modification refers to a chemical modification in which a methyl group is added to the nitrogen at the sixth position of adenosine in RNA molecules, making it the most prevalent, abundant, and conserved post-transcriptional modification in eukaryotic cells (13). This is widely present in both mRNAs and non-coding RNAs and mainly concentrated in coding sequences (CDS), 3′ untranslated regions (3′ UTR), long introns, and near stop codons (14). RRACH sequences are consensus motifs that are recognized and methylated by RNA methyltransferases (14, 15). In mRNA, m^6^A modifications are reversible and can be removed by demethylases. Reader proteins recognize specific m^6^A sites and regulate mRNA stability, localization, translation, splicing, and transport (13), and they interact to determine the fate of mRNA. m^6^A modifications are involved in the physiological and pathological processes of various immune cell types, playing crucial roles in cell differentiation, development, and disease pathogenesis (16). When macrophages polarize to the M1 phenotype, the expression of methyltransferase-like 3 (METTL3) and methyltransferase-like 14 (METTL14) is significantly upregulated, leading to m^6^A modification levels being markedly increase (17, 18). Recent studies indicate that m^6^A modifications are extensively involved in regulating macrophage phenotypes, further influencing disease progression by modulating metabolic pathways. In this paper, we are describing the role of m^6^A modification in macrophage metabolic reprogramming, including how this process alters metabolic disease progression, and we highlight potential opportunities for targeting m^6^A modifications to treat diseases.

## 2 m^6^A modification proteins in macrophage

m^6^A modification is a dynamic and reversible process controlled by three main types of proteins—writers, erasers, and readers (19)—as summarized in **Table 1**.

**Table 1:** Summary of m^6^A regulatory enzymes in macrophage polarization and inflammation.

Enzyme type	Enzyme name	Key genes targeted	Function in macrophages	Impact on polarization	Inflammatory effect	Disease association
Writers	METTL3	PGC-1α (20, 21)	METTL3 inhibits PGC-1α, contributing to mitochondrial dysfunction induced by oxLDL.	——	Pro-inflammatory	Atherosclerosis
		ApoC3 (22)	METTL3 promotes ApoC3 expression, leading to mitochondrial damage and enhancing calcium-dependent ROS generation.	——		ALI
		HDGF (23)	METTL3 facilitates HDGF expression, which drives aerobic glycolysis and mitochondrial damage in response to IFN-γ and LPS.	Pro-M1; anti-M2		Atherosclerosis
		DDIT4 (24)	METTL3 suppresses DDIT4, thereby promoting the activation of the mTORC1 and NF-κB signaling pathways.	——		NAFLD, obesity
		STAT1 (18, 25)	METTL3 upregulates the expression of STAT1.	Pro-M1; anti-M2		Atherosclerosis
		circN4bp (26)	Knockdown of METTL3 prevents the increase in circN4bp1 induced by LPS stimulation.	Pro-M1; anti-M2		Sepsis, ARDS
		TRAF6 (27)	METTL3 influences sympathetic remodeling and impacts the TRAF6/NF-κB pathway and oxidative stress levels.	——		Myocardial infarction
		NF-κ B (28, 29)	METTL3/METTL14 and NF-κB regulate each other.	Pro-M1		Atherosclerosis, NASH
		Braf (30)	METTL3 promotes Braf expression, activating the ERK signaling pathway.	Pro-M1; anti-M2		Atherosclerosis
		TTC4 (31)	METTL3 increases mitochondrial damage and ROS production by inhibiting the TTC4-HSP70 pathway.	——		Sepsis-induced ALI
		USP8 (32)	METTL3 activates pyroptosis through the METTL3/MALAT1/PTBP1/USP8/TAK1 axis.	——		Liver fibrosis
		pri-miR-34A (33)	METTL3 induces the maturation of miR-34a-5p, facilitating pyroptosis.	——		Alcoholic steatohepatitis
		hsa_circ_0029589 (34)	The METTL3/IRF-1 complex promotes pyroptosis by downregulating circ_0029589.	——		Acute coronary syndrome and atherosclerosis
		Trib1 (35)	METTL3 enhances Trib1 stability.	Pro-M2		Endometriosis
		PGP (36)	METTL3 inhibits PGP.	anti-M2		IBD
		SLC37A2 (37)	METTL3 promotes SLC37A2 expression through YTHDF1.	Pro-M2; anti-M1	Anti-inflammatory	IBD
		Pyk2 (38)	METTL3 ablation upregulates pyk2, activating the AKT/MAPKs signaling pathway.	——		——
		MSR1 (39)	METTL3 inhibits MSR1-induced lipid uptake.	——		Atherosclerosis
		PTX3 (40)	METTL3 suppresses the PTX3/STX17 signaling axis.	Pro-M1; anti-M2		Allergic asthma
		SOCS3 (41)	METTL3 upregulates SOCS3, inhibiting the JAK2-STAT3 pathway.	——		Syphilis
		NOD1/RIPK2 (42)	METTL3 knockdown activates the NOD1/RIPK2 signaling pathway.	Anti-M1		——
		SPRED2 (43)	METTL3 deletion inhibits YTHDF1-mediated SPRED2 translation, thereby promoting the ERK/STAT3 signaling pathway.	Anti-M1; anti-M2	Anti-tumor	B16
		pri-miR-146b (44)	METTL3 enhances miR-146b maturation and inhibits M2-type TAM polarization mediated by the PI3K/AKT signaling pathway.	Anti-M2		Colorectal cancer
		Irakm (17)	METTL3 promotes TLR4 signaling by inhibiting Irakm.	Pro-M1		Colon adenocarcinoma
	METTL14	Myd88 (45)	METTL14 boosts the NF-κB/IL-6 signaling pathway by promoting Myd88.	Pro-M1; anti-M2	Pro-inflammatory	Atherosclerosis
		NF-κB (28, 29)	METTL3/METTL14 and NF-κB mutually regulate activity.	Pro-M1		Atherosclerosis, NASH
		NLRP3 (46)	METTL14 mediates NLRP3 activation.	——		ALI, ARDS
		KAT3B (47)	METTL14 promotes KAT3B expression and induces pyroptosis via the H3K27ac-STING-NLRP3 pathway.	——		MCAO, OGD/R
		SOCS1 (48)	METTL14 maintains the negative feedback generated by SOCS1, inhibiting inflammatory pathways.	——	Anti-infection/inflammatory	Bacterial infection
		TSC1 (49)	METTL14 increases TSC1 expression, regulating the downstream MEK/ERK and C/EBPβ signaling pathways.	Pro-M2; anti-M1	Anti-inflammatory	UC
		SR-B1 (50)	METTL14 enhances SR-B1 expression.	——		Atherosclerosis
	WTAP	HIF-1α (51)	WTAP enhances the secretion of VEGF by promoting HIF-1α expression.	Pro-M1	Pro-inflammatory	Corneal neovascularization
		p65 (52)	WTAP mediates the nuclear translocation of p65 to regulate IL-6 expression.	——		——
Erasers	FTO	PPARγ (53)	FTO regulates cholesterol transport by inhibiting PPARγ expression.	——	Anti-inflammatory	Atherosclerosis
		ACSL4 (54)	FTO decreases ACSL4 mRNA stability via YTHDF1.	——		ALI
		Socs1 (48, 55)	FTO inhibits SOCS1.	——	Pro-infection/inflammatory	Bacterial infection
	ALKBH5	Slamf7 (56)	ALKBH5 downregulates Slamf7 and autophagy.	——	Pro-inflammatory	Silica-induced pulmonary inflammation
		SR-A5 (57)	ALKBH5 promotes the expression of SR-A5.	——	Anti-inflammatory	Sepsis
		CPT1A (58)	ALKBH5 enhances CPT1A expression.	Pro-M2		Colorectal cancer
Readers	HNRNPA2B1	pri-miR-146b (44)	HNRNPA2B1 promotes the maturation of miR-146b and inhibits M2-type TAM polarization mediated by the PI3K/AKT signaling pathway.	Anti-M2	Anti-tumor	Colorectal cancer
	YTHDC1	RHOH (59)	YTHDC1 promotes RHOH transcription.	——	Anti-inflammatory	IBD
	YTHDC2	TLR (60)	Knockdown of YTHDC2 after TM infection increases TLR2 expression.	——	Anti-infection	*Talaromyces marneffei*
		IFN-β (61)	ISG20 is activated by IFN-β early in viral infection, while YTHDC2 recruits ISG20 to degrade IFN-β mRNA later in the disease progression.	——	Pro-virus	Virus-infected
	YTHDF1	JAK2/STAT3 (62)	YTHDF1 knockdown inhibits the translation of phosphorylated proteins in the JAK2/STAT3 pathway.	——	Pro-inflammatory	Severe sepsis with ECMO
		NLRP3 (63)	Overexpression of YTHDF1 promotes the translation of NLRP3.	——		Sepsis
		Braf (30)	YTHDF1 facilitates the translation of Braf mRNA.	Pro-M1; anti-M2		Atherosclerosis
		SLC37A2 (37)	YTHDF1 promotes the translation of SLC37A2 mRNA.	Pro-M2; anti-M1	Anti-inflammatory	IBD
		ACSL4 (54)	YTHDF1 mediates ACSL4 mRNA degradation.	——		ALI
		p65 (64)	YTHDF1 specifically enhance p65 mRNA its translation.	——		Cerebral ischemia/reperfusion (I/R) injury
		SOCS3 (41)	YTHDF1 promotes SOCS3 mRNA translation and inhibits the JAK2/STAT3 pathway.	——		Syphilis
		NOD1 、RIPK2 (42)	YTHDF1 mediates the degradation of NOD1 and RIPK2 mRNAs, suppressing the NOD1/RIPK2 signaling pathway.	Anti-M1		——
		Socs1 (48, 55)	YTHDF1 stabilizes Socs1 mRNA.	——	Anti-infection/inflammatory	Bacterial infection
		SPRED2 (43)	YTHDF1 facilitates the translation of SPRED2, inhibiting the ERK/STAT3 signaling pathway.	Pro-M1; pro-M2	Pro-tumor	B16
	YTHDF2	PGC-1α (20, 21)	YTHDF2 mediates the degradation of PGC-1α mRNA.	——	Pro-inflammatory	——
		Pyk2 (38)	YTHDF2 facilitates the degradation of Pyk2 mRNA, inhibiting the AKT/MAPKs signaling pathways.	——	Anti-inflammatory	——
		NOD1、RIPK2 (42)	YTHDF2 mediates the degradation of NOD1 and RIPK2 mRNAs.	Anti-M1		——
		STAT1 (65)	YTHDF2 is involved in the degradation of STAT1 mRNA.	Anti-M1; pro-M2		——
	YTHDF3	PGP (36)	YTHDF3 promotes the translation of PGP mRNA.	Pro-M2	Anti-inflammatory	IBD
		PTX3 (40)	YTHDF3 mediates the degradation of PTX3 mRNA, inhibiting the PTX3/STX17 signaling axis.	Pro-M1; anti-M2		Allergic asthma
		FOXO3 (66)	YTHDF3 enhances the translation of FOXO3 mRNA and inhibits ISG.	——	Pro-virus	Virus-infected
	IGF2BP1	Gbp11、Cp (67)	IGF2BP1 stabilizes Gbp11 and Cp mRNAs.	——	Pro-inflammatory	Acute brain injury, chronic neurodegenerative diseases
	IGF2BP2	NLRP3 (46)	IGF2BP2 increases NLRP3 mRNA stability.	——		ALI, ARDS
		TSC1、PPARγ (68)	IGF2BP2 enhances the stability of TSC1 and PPARγ mRNAs.	Pro-M2; Anti-M1		Allergic lung inflammation
		TSC1、PPARγ (68)	IGF2BP2 enhances the stability of TSC1 and PPARγ mRNAs.	Pro-M2; Anti-M1	Anti-inflammatory	IBD
		YAP1 (69)	IGF2BP2 stabilizes YAP1 mRNA, influencing Hippo signaling regulation.	Pro-M2	Pro-tumor	NSCLC
		KLF12、c-MYC (70)	IGF2BP2 maintains the stability of KLF12 and c-MYC mRNAs.	Pro-M2		Pancreatic ductal adenocarcinoma
	IGF2BP3	MALAT1 (71)	hsa_circ_0004287 competes for IGF2BP3 binding, reducing MALAT1 stability, and thereby inhibiting MAPK phosphorylation.	——	Pro-inflammatory	Atopic dermatitis

### 2.1 Writers

m^6^A methyltransferases, such as METTL3, METTL14, Wilms’ tumor 1-associating protein (WTAP), methyltransferase-like 16 (METTL16), RNA-binding motif protein 15/15B (RBM15/15B), zinc finger CCCH domain-containing protein 13 (ZC3H13), HAKAI, zinc finger CCHC-type containing 4 (ZCCHC4), and vir-like m^6^A methyltransferase associated (VIRMA, KIAA1429), are writer proteins. METTL3, the first identified writer, is the only catalytically active subunit in the m^6^A methyltransferase complex (MTC) (72). In macrophages, METTL3 promotes glycolysis, mitochondrial damage, and ROS production while suppressing the TCA cycle and OXPHOS, thereby contributing to inflammatory responses. In diseases such as atherosclerosis, non-alcoholic steatohepatitis (NASH), non-alcoholic fatty liver disease (NAFLD), obesity, myocardial infarction, endometriosis, sepsis, acute lung injury (ALI), and inflammatory bowel disease (IBD), METTL3 promotes glycolysis (18, 23–25, 27–29, 35), enhances mitochondrial damage and ROS production (22, 31), and suppresses the TCA cycle and OXPHOS (20, 21, 36) by regulating the m^6^A modifications of key genes (see **Table 1** for details). Notably, M1 macrophages play a central role in driving inflammation and disease progression in conditions such as atherosclerosis, NASH, and sepsis (18, 23, 25, 26, 28–30). Also, METTL3 orchestrates these effects by reprogramming macrophage metabolism to favor M1 polarization and inflammatory responses.

The METTL3–METTL14 heterodimer, as the core of the MTC, catalyzes the majority of mRNA m^6^A methylation (72). Although METTL14 lacks catalytic activity, it structurally stabilizes the interaction between METTL3 and RNA substrates, enhancing the catalytic efficiency of the MTC (73). WTAP is a regulatory subunit that anchors METTL3 to METTL14 and facilitates the assembly and nuclear localization of the MTC (13). Both METTL14 and WTAP assist METTL3 in promoting M1 polarization and amplifying inflammatory responses. In diseases such as atherosclerosis, NASH and corneal neovascularization, METTL14 (28, 29, 45), and WTAP (51) enhance glycolysis, thereby promoting M1 polarization, inflammation, and disease progression.

While writers primarily enhance inflammation, they can also inhibit inflammatory processes (41, 42). In diseases such as IBD and atherosclerosis, writers can modulate the expression of solute carrier family 37 member 2 (SLC37A2), macrophage scavenger receptor 1 (MSR1), and scavenger receptor class B type 1 (SR-B1), inhibiting glycolysis and lipid uptake while promoting cholesterol efflux. This leads to anti-M1 and pro-M2 polarization, thereby suppressing macrophage inflammatory responses at the metabolic level (37, 39, 50). These findings suggest that macrophage m^6^A modifications are complex and multifaceted, contributing to both pro- and anti-inflammatory effects. The dual nature of this process makes it a “double-edged sword” for host immunity.

### 2.2 Erasers

“Erasers” are m^6^A demethylases, including fat mass and obesity-associated protein (FTO) and AlkB homolog 5 (ALKBH5). FTO was the first identified eraser (74). In diseases like atherosclerosis and ALI, FTO promotes fatty acid oxidation, inhibits lipid uptake, and accelerates cholesterol efflux, thereby facilitating M2 polarization and suppressing inflammation (53, 54, 68, 75). ALKBH5, the second identified m^6^A demethylase, shares a similar activity with FTO (19). It follows that m^6^A modifications dynamically modulate macrophage metabolic pathways in macrophages through the addition and removal of methyl groups by writers and erasers, balancing the pro-inflammatory M1 and anti-inflammatory M2 phenotypes.

### 2.3 Readers

“Readers” are m^6^A-specific reader proteins, including YTH domain containing proteins 1/2 (YTHDC1/2), heterogeneous nuclear ribonucleoproteins (hnRNPs), insulin-like growth factor 2 mRNA-binding protein 1/2/3 (IGF2BP1/2/3), and YTH domain family proteins 1/2/3 (YTHDF1/2/3). The nuclear m^6^A reader binds m^6^A-containing precursor RNAs in the nucleus and participates in RNA-selective shearing. Intranuclear m^6^A readers include YTHDC1 and hnRNP family members (13). YTHDC2, an RNA helicase, aids in RNA binding and influences mRNA translation or degradation (76). After being processed from the precursor transcript, mature mRNAs are further regulated by cytoplasmic m^6^A readers. YTHDF1 promotes mRNA translation, YTHDF2 facilitates mRNA degradation, and YTHDF3 contributes to either process (77). The IGF2BP family, which includes IGF2BP1, IGF2BP2, and IGF2BP3, stabilizes mRNA (78).

While the dynamic m^6^A levels are regulated by writers and erasers, m^6^A-modified target mRNAs are primarily regulated by readers. For example, YTHDF1 and YTHDF2 are required for the inhibitory effect of METTL3/METTL14 on suppressor of cytokine signaling 3 (SOCS3), nucleotide-binding oligomerization domain containing 1 (NOD1), and receptor interacting protein kinase 2 (RIPK2)-mediated inflammation (41, 42). These studies indicate that the role of readers in macrophage regulation is dependent on whether the target genes are pro- or anti-inflammatory. The role of readers in macrophage regulation is complex, as it depends not only on the nature of the target genes (pro- or anti-inflammatory) but also on the disease context. Even within the same macrophage mechanism, readers can exhibit different inflammatory effects depending on the disease. For example, IGF2BP2 enhances the stability of tuberous sclerosis complex 1 (TSC1) and peroxisome proliferator-activated receptor gamma (PPAR-γ) mRNAs, promoting an anti-inflammatory response in IBD but a pro-inflammatory response in allergic lung inflammation (68).

## 3 Role of m^6^A in macrophage metabolism

Macrophage metabolic reprogramming involves dynamic processes such as glycolysis, the TCA cycle, OXPHOS, FAO, and cholesterol transport, with the specific roles of m^6^A modifiers in these pathways summarized in **Table 2**.

**Table 2:** Summary of m^6^A modifiers and their specific roles in macrophage metabolism.

Enzyme type	Enzyme name	Key genes targeted	Effect on macrophage metabolism	Disease association
Writers	METTL3	HDGF (23)	METTL3 facilitates HDGF expression, leading to enhanced glycolysis and mitochondrial dysfunction.	Atherosclerosis
		DDIT4 (24)	METTL3 suppresses DDIT4 expression, thereby promoting the activation of the mTORC1 and NF-κB signaling pathways, which subsequently drive HIF-1α-mediated glycolysis.	NAFLD, obesity
		STAT1 (18, 25)	METTL3 upregulates STAT1 expression, enhancing glycolysis.	Atherosclerosis
		TRAF6 (27)	METTL3 regulates sympathetic remodeling and affects the TRAF6/NF-κB pathway. Through NF-κB activation, it regulates HIF-1α expression, driving glycolysis.	Myocardial infarction
		NF-κ B (28, 29)	The METTL3/METTL14 complex enhances NF-κB expression, which, in turn, regulates HIF-1α to drive glycolysis.	Atherosclerosis, NASH
		Trib1 (35)	METTL3 enhances Trib1 stability, which promotes glycolysis through the ERK/STAT3 signaling pathway.	Endometriosis
		PGC-1α (20, 21)	METTL3 inhibits PGC-1α expression with the assistance of YTHDF2, contributing to mitochondrial dysfunction and suppressing OXPHOS.	Atherosclerosis
		PGP (36)	METTL3 knockout increases YTHDF3-mediated PGP expression, thereby promoting the TCA cycle, OXPHOS, and PPP.	IBD
		ApoC3 (22)	METTL3 promotes ApoC3 expression, resulting in mitochondrial damage and enhanced calcium-dependent ROS generation.	ALI
		TTC4 (31)	METTL3 increases mitochondrial damage and ROS production by inhibiting the TTC4-HSP70 pathway.	Sepsis-induced ALI
		SLC37A2 (37)	METTL3 promotes SLC37A2 expression via YTHDF1, where SLC37A2 suppresses glycolysis.	IBD
		MSR1 (39)	METTL3 inhibits MSR1-induced lipid uptake.	Atherosclerosis
	METTL14	Myd88 (45)	METTL14 boosts the NF-κB/IL-6 signaling pathway by promoting MyD88 expression. NF-κB further regulates HIF-1α activity, enhancing glycolysis.	Atherosclerosis
		NF-κB (28, 29)	METTL3/METTL14 and NF-κB mutually regulate each other’s activity, with NF-κB further modulating HIF-1α activity to drive glycolysis.	Atherosclerosis, NASH
		SR-B1 (50)	METTL14 enhances SR-B1 expression, thereby facilitating cholesterol efflux.	Atherosclerosis
	WTAP	HIF-1α (51)	WTAP enhances HIF-1α translation efficiency, thereby driving glycolysis.	Corneal neovascularization
		NF-κB p65 (52)	WTAP mediates the nuclear translocation of NF-κB p65, which further regulates HIF-1α activity and enhances glycolysis.	——
Erasers	FTO	PPARγ (75) (53)	FTO regulates cholesterol transport by inhibiting PPARγ expression. FTO knockout reduces PPARγ mRNA stability through YTHDF2, thereby suppressing FAO.	Atherosclerosis
		ABCA1\ABCG1 (53)	FTO enhances cholesterol efflux by phosphorylating AMPK and increasing ABCA1 and ABCG1 expression.	Atherosclerosis
		ACSL4 (54)	FTO decreases ACSL4 mRNA stability via YTHDF1, thereby promoting FAO.	ALI
	ALKBH5	SR-A5 (57)	The TCA cycle intermediate α-KG upregulates ALKBH5, promoting SR-A5 expression.	Sepsis
		CPT1A (58)	ALKBH5 enhances CPT1A expression, thereby promoting FAO and mitochondrial respiration.	Colorectal cancer
Readers	YTHDF1	SLC37A2 (37)	YTHDF1 promotes the translation of SLC37A2 mRNA, which suppresses glycolysis.	IBD
		ACSL4 (54)	YTHDF1 mediates ACSL4 mRNA degradation, thereby promoting FAO.	ALI
	YTHDF2	PGC-1α (20, 21)	YTHDF2 mediates the degradation of PGC-1α mRNA, contributing to mitochondrial dysfunction and suppressing OXPHOS.	Atherosclerosis
		PPARγ (75)	YTHDF2 reduces PPARγ mRNA stability and expression, thereby inhibiting FAO.	——
		STAT1 (65)	YTHDF2 is involved in the degradation of STAT1 mRNA, leading to the suppression of glycolysis.	——
	YTHDF3	PGP (36)	YTHDF3 promotes the translation of PGP mRNA, which enhances the TCA cycle, OXPHOS, and PPP.	IBD
	IGF2BP2	TSC1、PPARγ (68)	IGF2BP2 enhances the stability of TSC1 and PPARγ mRNAs. PPARγ promotes fatty acid oxidation and OXPHOS, while TSC1 suppresses glycolysis and promotes OXPHOS by inhibiting mTORC1.	Allergic lung inflammation/IBD
		KLF12、c-MYC (68)	IGF2BP2 maintains the stability of KLF12 and c-MYC mRNAs, regulating glycolysis through c-MYC.	Pancreatic ductal adenocarcinoma

### 3.1 Glycolysis

**Figure 2:**
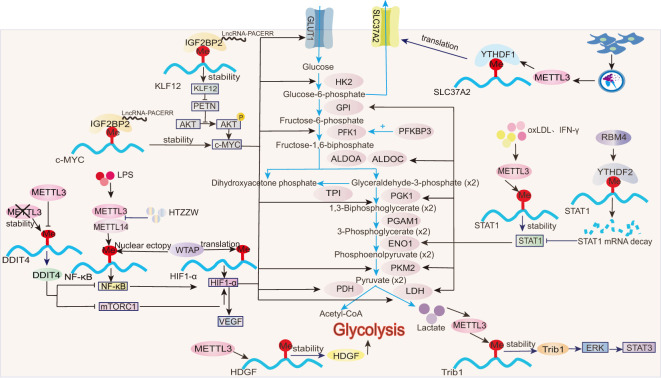
Role of m^6^A in glycolysis. In macrophages, m^6^A modifications influence the stability, translation, and expression of transcription factors (e.g., HIF-1α, STAT1), then modulating glycolytic enzymes (e.g., HK2, PKM2).

The activation of classical M1 macrophages is characterized by an upregulation of glycolysis, which is accompanied by an increased extracellular acidification rate (ECAR) and a decreased oxygen consumption rate (OCR) (79). This shift ultimately results in mitochondrial dysfunction, including reduced oxygen uptake and ATP production (80). M1 macrophages become activated by stimulating bone-marrow-derived macrophages with LPS for 24 h. This leads to a significant upregulation of the glucose transporter solute carrier family 2 member 1 (SLC2A1, also known as GLUT1) and the glycolytic enzymes, hexokinase 3 (HK3), 6-phosphofructo-2-kinase/fructose-2,6-bisphosphatase 3 (PFKFB3), phosphoglucomutase 2 (PGM2), and alpha-enolase (ENO2), along with an accumulation of the glycolytic product, lactate (81). See **Figure 2** for additional glycolytic enzyme and product changes. GLUT1 is the primary glucose transporter in mouse macrophages and is upregulated 10-fold and twofold in M1 and M2 macrophages, respectively, after polarization from M0 cells (4). Macrophages use GLUT1 to take up glucose in response to LPS stimulation (82). ECAR represents the rate of glycolysis, while OCR indicates mitochondrial respiration. GLUT1 overexpression in macrophages effectively increases ECAR, reduces OCR, and promotes macrophage polarization toward the M1 inflammatory phenotype (4). All m6A-regulated mechanisms of macrophage glycolysis are summarized in **Figure 2**. During atherosclerosis, METTL3 regulates hepatoma-derived growth factor (HDGF) mRNA stability in macrophages through m^6^A modification, increasing HDGF expression that, in turn, accelerates glycolysis and enhances mitochondrial dysfunction. This is reflected in increased glucose uptake and ECAR, decreased OCR, and reduced mitochondrial membrane potential, thereby promoting M1 macrophage polarization (23).

Signal transducer and activator of transcription 1 (STAT1) is a hallmark transcription factor involved in M1 macrophage polarization. After stimulation with IFN-γ or oxLDL, m^6^A modification increases significantly in macrophages. METTL3 directly mediates m^6^A modifications on the CDS and 3′UTR regions of STAT1 mRNA, thereby enhancing the stability, gene expression, and transcriptional activity of STAT1 (18, 25). STAT1, acting at both the transcriptional and translational levels, upregulates key glycolytic enzymes, including glucose-6-phosphate isomerase (GPI), fructose-bisphosphate aldolase A (ALDOA), fructose-bisphosphate aldolase C(ALDOC), triosephosphate isomerase (TPI), phosphoglycerate kinase 1 (PGK1), phosphoglycerate mutase 1 (PGAM1), ENO1, pyruvate kinase M2 isoform (PKM2), and lactate dehydrogenase (LDH). Furthermore, STAT1 promotes the upregulation of NADH, FADH2, and ATP synthase to meet the increased energy demands of M1 macrophages during polarization (83). In RAW264.7 macrophages, overexpression of RNA-binding motif 4 (RBM4) promotes the binding of the reader protein YTHDF2 to m^6^A sites on the STAT1 mRNA 3′UTR region, inducing STAT1 mRNA degradation and reducing STAT1 transcriptional activity. Consequently, the expression of glycolysis-related genes regulated by STAT1, such as ALDOA, ALDOC, and GPI, is significantly decreased (65). This suppression of glycolysis is reflected by decreased glucose uptake, reduced lactate production, and downregulated ECAR profiles (65). Notably, overexpression of RBM4 inhibits IFN-γ-induced M1 macrophage polarization without affecting IL-4-induced M2 polarization, suggesting a selective regulatory role in metabolic reprogramming (65).

IL-4-induced M2 macrophage activation is primarily dependent on mitochondrial respiratory function. However, this process is also accompanied by a moderate enhancement in glycolysis, as indicated by delayed and slight increases in ECAR (79). While glycolysis is well established as a key metabolic feature of M1 macrophage polarization, emerging evidence suggests its involvement in M2 polarization under specific contexts. For instance, in endometriosis, enhanced glycolysis is positively associated with the infiltration of M2 macrophages into lesion sites, as evidenced by the increased levels of PKM2 and lactate (35). High lactate concentrations promote the stability of tribbles pseudokinase 1 (Trib1) mRNA through METTL3-mediated m^6^A modifications, thereby increasing Trib1 protein expression (35). Trib1 facilitates M2 macrophage polarization through the activation of the ERK/STAT3 signaling pathway (35, 84). Collectively, glycolysis may contribute to M2 macrophage activation in some contexts (35, 65), but its precise role depends on the specific inflammatory immune environment.

Glucose-6-phosphate (G6P) is an intermediate in glycolysis. SLC37A2, a phosphate-linked G6P antiporter, exhibits the highest transcript abundance among the SLC37 family in macrophages. LPS-induced glycolysis is increased in SLC37A2 knockout macrophages, characterized by the rapid depletion of key glycolytic intermediates such as G6P and dihydroxyacetone phosphate (DHAP), alongside the increased accumulation of downstream products, including pyruvate and lactate. This metabolic shift is accompanied by elevated ECAR (85). Notably, SLC37A2 expression is significantly reduced in the colorectal tissues of IBD mouse models (37). In RAW264.7 macrophages, exosomes derived from human umbilical cord mesenchymal stem cells (hucMSC-Ex) mediate the m^6^A modification of SLC37A2 3′UTR mRNA through METTL3 and enhance the binding of the reader protein YTHDF1 to SLC37A2, thereby promoting SLC37A2 mRNA translation and expression (37). By downregulating glycolysis, SLC37A2 mitigates macrophage inflammatory activation, thereby alleviating intestinal inflammation in IBD (37).

Hypoxia-inducible factor 1 α (HIF-1α) is a key regulator of glycolysis (86). The rate-limiting enzyme of glycolysis, pyruvate kinase isozyme type M 2 (PKM2), is upregulated in LPS-treated macrophages, and PKM2 dimers promote HIF-1α stabilization (87). HIF-1α drives glycolysis by inducing the expression of several genes, including GLUT1, hexokinase 2 (HK2), phosphofructokinase 1 (PFK1), LDH, and pyruvate dehydrogenase (PDH) (81, 86). In corneal neovascularization, the methyltransferase, WTAP, modulates the m^6^A enrichment level on HIF-1α and partially affects its translation efficiency without changing its mRNA stability, thereby promoting HIF-1α protein production (51).

LPS enhances HIF-1α mRNA transcription in macrophages by inducing nuclear factor kappa B (NF-κB) activity, further amplifying HIF pathway signaling (88, 89). Beyond its role in HIF-1α translation (51), WTAP also facilitates the nuclear translocation of NF-κB p65 in tamm-horsfall protein-1 (THP-1) macrophages (52), enhancing HIF-1α stability and activity via NF-κB-dependent mechanisms (88, 89). In LPS-stimulated macrophages, NF-κB recruits the RelA subunit (NF-κB p65) to the HIF-1α promoter to regulate its transcription (89). In BMDMs treated with the hypoxia mimetic drug deferoxamine (DFX), IκB kinase β (IKKβ) is indispensable for HIF-1α accumulation (89). In the absence of IKKβ, hypoxia-driven HIF-1α expression and the transcription of its downstream targets, such as vascular endothelial growth factor (VEGF) and GLUT1, are significantly impaired (89). In LPS-stimulated liver macrophages (Kupffer cells), the NF-κB p65 subunit transcriptionally activates METTL3 and METTL14 and increases global m^6^A modification levels (29). The traditional Chinese herbal formula, Huatuo Zizao pill (HTZZW), mitigates atherosclerosis by attenuating NF-κB signaling. Specifically, HTZZW suppresses METTL3 and METTL14 expression in macrophages, reducing m^6^A modification within the NF-κB mRNA 3′-UTR and ultimately lowering NF-κB mRNA translation and protein expression (28). m^6^A modification influences NF-κB activity through the methyltransferases, METTL3 and METTL14, which, in turn, regulates HIF-1α through NF-κB, driving the expression of glycolytic genes and significantly promoting M1 macrophage polarization.

In addition to NF-κB, the mechanistic target of rapamycin complex 1 (mTORC1) also serves as an upstream regulator of HIF-1α, inducing the transcription of glycolytic genes such as LDH and PGK1 as well as VEGF (90). In METTL3-deficient macrophages, the stability of DNA damage-inducible transcript 4 (DDIT4) mRNA is significantly enhanced (24). DDIT4 inhibits mTORC1 and NF-κB signaling pathways, thereby reducing downstream HIF-1α transcriptional activity. This suppression reduces glycolysis and M1 macrophage activation, ultimately protecting against diet-induced nonalcoholic fatty liver disease (NAFLD) and obesity (24).

MYC oncogene (c-MYC) is also a core regulator of glycolysis (91, 92). In pancreatic ductal adenocarcinoma (PDAC), the reader IGF2BP2 interacts with LncRNA-PACERR to enhance the stability and expression of krüppel-like factor 12 (KLF12) and c-MYC mRNAs. This interaction further increases c-MYC expression through KLF12\PETN\pAKT signaling (70). c-MYC overexpression significantly increases the expression of GLUT1, SLC1A5, and the glycolytic enzymes HK2, PKM2, and LDH, promoting the M2 polarization of tumor-associated macrophages (TAMs) (70, 91, 92).

### 3.2 TCA cycle

**Figure 3:**
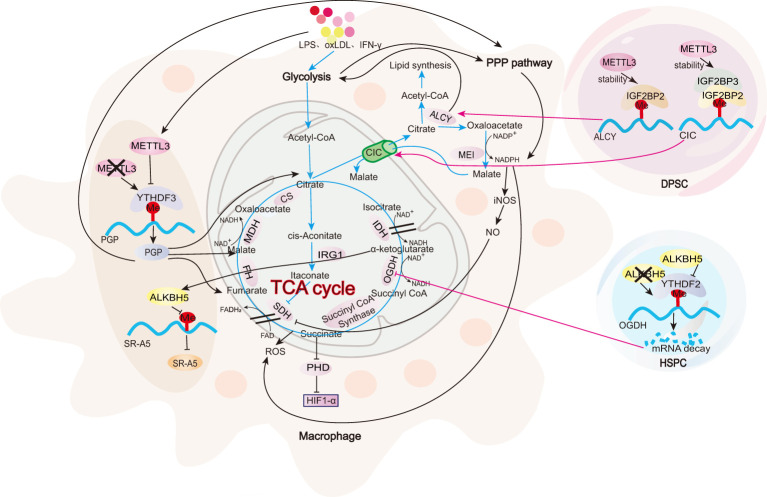
Role of m^6^A in TCA cycle. In macrophages, m^6^A modifications regulate PGP, which, in turn, modulates key metabolic enzymes and intermediates involved in the TCA cycle. Moreover, DPSCs and HSPCs serve as examples to illustrate the impact of m^6^A modifications on two critical metabolic checkpoints: citrate metabolism and succinate metabolism. Further investigation is needed to explore these checkpoints in the context of macrophages.

All m6A-regulated mechanisms of TCA cycle in macrophages are summarized in **Figure 3**. The TCA cycle remains intact in M2 macrophages (93). In colonic infiltrating macrophages from METTL3-knockout IBD mice, YTHDF3-mediated upregulation of phosphoglycolate phosphatase (PGP) mRNA and protein expression has been observed (36). PGP enhances the TCA cycle, OXPHOS, and the pentose phosphate pathway (PPP) in macrophages, while glycolysis remains unaffected (36). This is evidenced by the increased levels of TCA intermediates such as malate, citrate, and fumarate along with elevated OCR. In addition, the PPP-related genes, including glucose-6-phosphate dehydrogenase X (G6PDX), phosphogluconate dehydrogenase (PGD), and 6-phosphogluconolactonase (PGLS), are markedly upregulated, leading to an increased NADPH/NADP+ ratio. Conversely, lactate levels, ECAR, and expression of the glycolytic enzymes remain unchanged (36). The metabolic reprogramming in METTL3-deficient macrophages promotes M2 polarization, suppressing pathogenic Th1 cells and alleviating colitis (36).

In M1 macrophages, alongside the upregulation of glycolysis, there is a marked reduction in the levels of key mitochondrial enzymes, such as malate dehydrogenase (MDH1) and isocitrate dehydrogenase (IDH2). This reduction, coupled with a significant depletion of isocitrate in the mitochondrial respiratory chain, indicates an impairment of the TCA cycle (81). The disruption of the TCA cycle occurs at two key metabolic points: citrate metabolism and succinate metabolism (94).

During citrate metabolism, LPS-, TNF-α-, or IFN-γ-activated M1 macrophages show a reduction in IDH expression (95), leading to isocitrate depletion in the mitochondria. At the same time, the mitochondrial citrate carrier (CIC, also known as solute carrier family 25 member 1, SLC25A1) is upregulated, facilitating the export of citrate from the mitochondria to the cytoplasm in exchange for malate. This results in the accumulation of citrate in the cytoplasm (96, 97), where ATP-citrate lyase (ACLY) converts it into acetyl-CoA and oxaloacetate (98). The increase in acetyl-CoA promotes lipid synthesis, while oxaloacetate is converted into malate by malic enzyme (ME1). This is accompanied by the reduction of NADP^+^ to NADPH, enhancing the production of nitric oxide (NO) and ROS (99). m^6^A modification at this first interruption point in the TCA cycle has been identified in dental pulp stem cells (DPSCs). In these cells, METTL3-IGF2BP2 mediates the stability of ACLY mRNA, while METTL3-IGF2BP2/3 mediates the stability of CIC mRNA (100). Additional research is required to validate this observation in macrophages.

During succinate metabolism, activated M1 macrophages show a significant upregulation of aconitate decarboxylase 1 (ACOD1, also known as IRG1) (101). ACOD1 catalyzes the decarboxylation of cis-aconitate to produce itaconate, thereby disrupting the TCA cycle (101). Itaconate inhibits succinate dehydrogenase (SDH), preventing succinate from further metabolizing into fumarate and leading to its accumulation in the cell (102). Succinate acts as a competitive inhibitor of α-ketoglutarate (α-KG), suppressing the activity of HIF-1α hydroxylase (PHD) (81) and stabilizing and activating HIF-1α, a key transcription factor for glycolysis (103). The m^6^A modification at this second interruption point in the TCA cycle has been observed in hematopoietic stem and progenitor cells (HSPCs). In these cells, the α-ketoglutarate dehydrogenase complex (OGDH), the rate-limiting enzyme in the conversion of α-KG to succinyl-CoA, is regulated by the m^6^A demethylase ALKBH5. Deletion of ALKBH5 increases m^6^A modifications on OGDH mRNA, which are recognized by the m^6^A reader protein YTHDF2. This recognition reduces OGDH mRNA stability, decreases OGDH protein levels, and consequently slows TCA cycle progression (104). In macrophages, α-KG upregulates the m^6^A demethylase, ALKBH5, reducing the m^6^A modification of scavenger receptor class A member 5 (SR-A5), enhancing SR-A5 expression, inhibiting M1 polarization, and promoting M2 polarization, thereby alleviating the inflammatory response in septic macrophages (57).

### 3.3 OXPHOS

**Figure 4:**
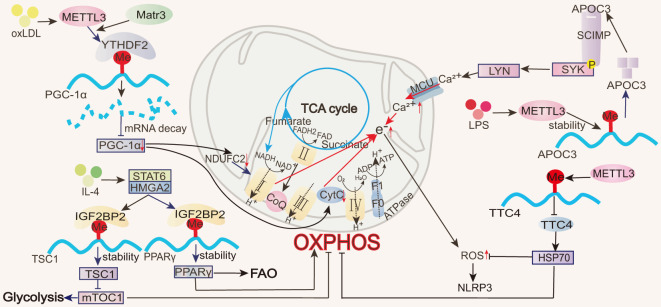
Role of m^6^A in OXPHOS. In macrophages, m^6^A modifications regulate transcription factors (e.g., PGC-1α, PPARγ), thereby affecting the electron transport chain in OXPHOS and influencing ROS production.

IL-4-induced M2 macrophages are characterized by enhanced oxidative metabolism, exhibiting high levels of OCR and spare respiratory capacity (SRC) (10) and primarily relying on OXPHOS and FAO (9). All m6A-regulated mechanisms of macrophage OXPHOS are summarized in **Figure 4**. In response to IL-4, STAT6 directly binds to the high-mobility group AT-hook 2 (HMGA2) promoter region, promoting the expression of the reader IGF2BP2 (68). IGF2BP2 enhances TSC1 and PPARγ mRNA stability and expression in an m^6^A modification-dependent manner (68). Among them, PPARγ promotes FAO and OXPHOS (105), while TSC1 inhibits glycolysis and induces OXPHOS (90, 106). PPARγ and TSC1 expression effectively elevates OCR and SRC production, enhancing OXPHOS and mitochondrial respiration and promoting M2 cell polarization (68). This process not only exacerbates allergic pulmonary inflammation but also has a positive role in alleviating colitis (68).

NADH and FADH2, generated during the TCA cycle, are critical electron donors for OXPHOS. During M1 macrophage activation, TCA cycle disruption leads to succinate accumulation, causing reverse electron transfer (RET) in the mitochondria. This increases electron leakage in the electron transport chain, reducing OXPHOS efficiency and generating ROS (107). Excessive ROS can impair mitochondrial function, favoring a shift from OXPHOS to glycolysis for ATP production. During ALI, METTL3 modulates the stability and expression of apolipoprotein C3 (ApoC3) mRNA in macrophages. ApoC3, through the SCIMP-SYK signaling pathway, promotes calcium influx, damages the mitochondrial membrane potential, impairs OXPHOS, generates ROS and activates the NOD-like receptor family pyrin domain containing 3 (NLRP3) inflammasome (22). METTL3 also suppresses the stability and expression of tetratricopeptide repeat domain 4 (TTC4) 3’-UTR mRNA in ALI macrophages, inhibiting downstream heat shock protein 70 (HSP70) induction, increasing mitochondrial damage, and activating the ROS/NLRP3 signaling pathway (31).

Peroxisome proliferator-activated receptor gamma coactivator 1-alpha (PGC-1α) is an important regulator of OXPHOS, binding to various transcription factors and nuclear hormone receptors to support mitochondrial biogenesis and oxidative metabolism. This includes the activation of nuclear respiratory factor 1 (NRF-1), estrogen-related receptor alpha (ERRα), yin yang 1 (YY1), and myocyte enhancer factor 2C (MEF2C), which act on the mitochondrial respiratory chain, and PPARα and PPARγ, which control FAO (108). During oxLDL-induced monocyte inflammation, METTL3 mediates m^6^A modifications in the coding region of PGC-1α. YTHDF2 specifically recognizes these m^6^A modifications and promotes the degradation of PGC-1α mRNA, reducing the PGC-1α levels (20, 21). The reduction in PGC-1α further inhibits the expression of the nuclear-encoded mitochondrial respiratory chain proteins, cytochrome c somatic (CYCS), and NADH: ubiquinone oxidoreductase core subunit C2 (NDUFC2), thereby suppressing OXPHOS, increasing the accumulation of cellular and mitochondrial ROS, reducing OCR, and impairing mitochondrial function (20, 21). Matr3 participates in the formation of the METTL3–METTL14 complex. While Matr3 overexpression does not directly affect the level of m^6^A in polyadenylated RNA, this protein can assist METTL3 in increasing the m^6^A modifications of PGC-1α mRNA (20, 21).

### 3.4 FAO

**Figure 5:**
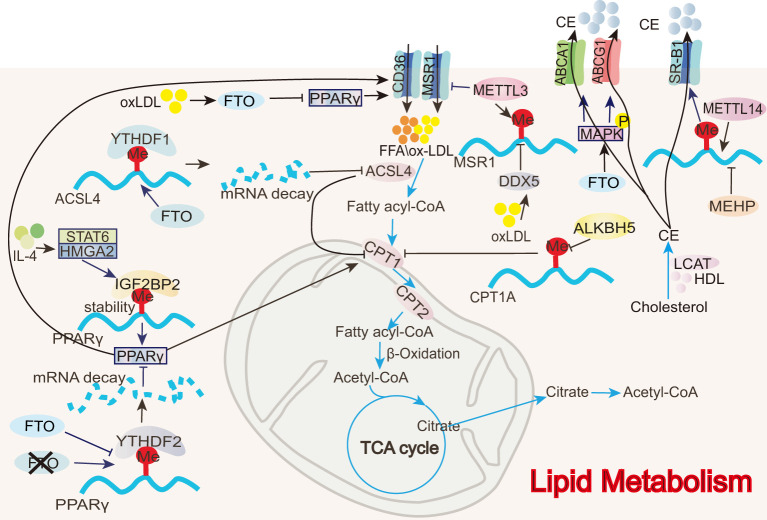
Role of m^6^A in FAO and cholesterol transport. In macrophages, m^6^A modifications directly or indirectly regulate FAO-related enzymes (e.g., ACSL4, CPT1) as well as lipid transport proteins (e.g., ABCA1, ABCG1).

All m6A-regulated mechanisms of FAO in macrophages are summarized in **Figure 5**. Oxidative metabolism in M2 macrophages primarily depends on FAO and OXPHOS (9). The rate-limiting step of FAO is mediated by carnitine palmitoyltransferase 1A (CPTIA), which converts long-chain fatty acids into acyl-carnitine, facilitating its transport into the mitochondrial matrix (109) and making CPT1A the most critical transport enzyme for FAO (110). FAO and ATP levels increase significantly when THP-1 macrophages are co-cultured with colorectal cancer (CRC) cells. This is accompanied by a notable increase in OCR and a decrease in ECAR, indicating that fatty acid metabolism and mitochondrial respiration are increased in the macrophages, inducing TAM M2 polarization and promoting tumor growth (58). Notably, ALKBH5 mediates a reduction in the m^6^A modification of CPT1A, increasing CPT1A mRNA stability and expression and thus regulating the FAO pathway involved in M2 polarization (58).

Acyl-CoA synthetase long-chain family member 4 (ACSL4) is involved in polyunsaturated fatty acid metabolism, promoting lipid peroxide production during fatty acid oxidation (111). Inhibiting ACSL4 enhances mitochondrial respiration and fatty acid oxidation, as evidenced by increased OCR, mitochondrial membrane potential, ATP production, and a higher expression of genes, including PPARα, PGC1α, CPT1A, acetyl-CoA carboxylase 2 (ACC2), and acyl-CoA dehydrogenase long-chain (ACADL), associated with mitochondrial fatty acid oxidation (111). During ALI, FTO reduces the stability of ACSL4 mRNA via YTHDF1, interrupting poly-unsaturated fatty acid metabolism, decreasing lipid peroxide production, and promoting FAO to suppress macrophage inflammation and inhibit disease progression (54). The FTO inhibitor FB23-2 exacerbates lung injury and inflammation in ALI mice (54).

Peroxisome proliferator-activated receptor gamma (PPARγ) enhances fatty acid uptake by upregulating CD36 expression and accelerates fatty acid entry into the mitochondria by promoting CPT1 activity, thereby increasing FAO (105). m^6^A modifications regulate PPARγ expression, modulating the FAO process and impacting M2 macrophage polarization. In BMDMs, IGF2BP2 recognizes the m^6^A modification on PPARγ mRNA and enhances its stability and expression, thus promoting M2 polarization (68). In contrast, an FTO gene knockout reduces PPARγ mRNA stability and expression through YTHDF2 involvement, thereby hindering macrophage M2 polarization (75).

### 3.5 Cholesterol transport

All m6A-regulated mechanisms of cholesterol transport in macrophages are summarized in **Figure 5**. CD36 is a transmembrane transport protein widely expressed in various tissues, including adipose tissue, heart, and skeletal muscle, and it is responsible for the uptake and transport of fatty acids and cholesterol (53, 112). Low-density lipoprotein (LDL) is a lipoprotein particle that carries cholesterol into peripheral tissue cells. Upon oxidization, LDL forms oxidized low-density lipoprotein (oxLDL) (113). CD36 facilitates the uptake of oxLDL by macrophages, leading to the formation of foam cells, which can induce inflammatory responses and contribute to atherosclerosis (53). In RAW 264.7 macrophages, the eraser FTO inhibits PPARγ expression, suppressing CD36 uptake of oxLDL and preventing foam cell formation (53). Like CD36, MSR1 is a scavenger receptor that can also take up oxLDL and contribute to foam cell formation (39). oxLDL promotes dead-box helicase 5 (DDX5) expression in THP-1 macrophages. DDX5 then inhibits METTL3 methyltransferase activity, reducing m^6^A modifications and thus stabilizing MSR1 mRNA, increasing MSR1 expression, and inducing lipid uptake and atherosclerosis (39).

Reverse cholesterol transport (RCT) is the process by which cholesterol from lipid-laden peripheral cells is transported to the liver via plasma high-density lipoprotein (HDL) and subsequently excreted from the body through bile acids (114). Unlike lipid uptake, RCT can inhibit the formation of macrophage foam cells and the progression of atherosclerosis. RCT can be mediated by three pathways: simple diffusion, scavenger receptor B1 (SR-B1)-mediated facilitated diffusion, and efflux mediated by ATP-binding cassette transporter A1 (ABCA1) or G1 (ABCG1) (114). In mono- (2-ethylhexyl) phthalate (MEHP)-exposed RAW 264.7 macrophages, m^6^A RNA methylation is significantly reduced. MEHP inhibits SR-B1 expression by decreasing METTL14 expression, suppressing cholesterol efflux from macrophages, and accelerating atherosclerosis (50). In contrast, FTO increases ABCA1 and ABCG1 expression by phosphorylating AMPK, thereby enhancing cholesterol efflux and hindering disease progression (53).

## 4 Current status of m^6^A modification-based treatment options

Recently, m^6^A modification has played a significant role in the characterization of macrophage metabolic reprogramming and has been identified as an important therapeutic target for macrophage-related inflammatory diseases. Drugs that directly affect m^6^A modification or the activity of m^6^A-modifying proteins are used to modulate macrophage activation, polarization, and inflammatory responses. For example, Hua Tuo Zai Zao Wan (HTZZW) reduces the m^6^A levels in macrophages by inhibiting METTL3 and METTL14, leading to destabilization and downregulation of NF-κB mRNA, which suppresses macrophage polarization toward the M1 phenotype, effectively alleviating atherosclerosis (28). Coptisine (COP) increases macrophage m^6^A methylation by upregulating METTL14, enhancing TSC1 mRNA stability, inhibiting M1 polarization, and promoting M2 polarization, effectively alleviating ulcerative colitis (49). Astragalus mongholicus polysaccharides (APS) counteract the LPS-induced elevation of m^6^A levels in macrophages by inhibiting WTAP. This prevents WTAP-mediated p65 nuclear translocation, reducing IL-6 expression and alleviating macrophage-mediated inflammation (52).

Moreover, several specially designed small-molecule inhibitors, which exhibit good specificity and regulatory properties, are easy to synthesize and modify, showing promising translational potential in macrophage-related inflammatory diseases. For instance, the METTL3 inhibitor STM2457 suppresses the inflammatory response of M1 macrophages by inhibiting the LPS-induced NF-κB signaling pathway, thereby reducing the incidence of bone marrow inflammation in mice (115). The METTL3 inhibitors F039-0002 and 7460-0250 specifically inhibit METTL3 activity in macrophages, regulating glucose metabolic reprogramming and significantly alleviating intestinal inflammation (36). In lung macrophages (MH-S), treatment with the METTL3 inhibitor 3-deazaadenosine (DAA) nearly restores the elevated levels of circN4bp1 induced by LPS stimulation to normal, inhibiting M1 macrophage activation and improving the prognosis of septic patients (26). Conversely, the FTO inhibitor FB23-2 exacerbates the inflammatory response, lung injury, and iron dysregulation in ALI mice (54). Other METTL3 inhibitors include UZH1a and UZH1b (116), RM3, and RSM3 (117); other FTO inhibitors include CS1 and CS2 (118), R-2HG (119), and MA (120); and other ALKBH5 inhibitors include IOX3 (121), Ena15 and Ena21 (122), Cpd 20m (123), and DDO-2728 (124) have also been developed. The efficacy of these inhibitors in treating macrophage-related inflammatory diseases requires further investigation.

While small molecule inhibitors have a high potential for clinical translation, their long-term safety and side effects require additional validation. Nanoparticle (NP) drug delivery systems may help to enhance small molecule inhibitor specificity and safety. For example, red blood cell microvesicles can deliver STM2457 to activated monocytes, inhibiting NF-κB signaling-specific inflammation. This method is used to treat monocyte inflammation and fibrosis related to cardiac remodeling associated with device implantation (125).

miRNAs and siRNAs are also delivered by NPs to alter macrophage activation and polarization. For instance, miR-1208 in hucMSCs-EVs regulates the m^6^A levels of NLRP3 mRNA, preventing the activation of the NLRP3 inflammasome and inhibiting pro-inflammatory factors in macrophages, thereby slowing the progression of knee osteoarthritis (126). NPs can also encapsulate METTL3, METTL14, SPRED2 mRNA, or IRAKM siRNA, specifically targeting TAMs and reprogramming them from an M2 to an M1 phenotype, thus remodeling the tumor microenvironment (TME) (127).

## 5 Conclusion

This article reviews recent findings on the role of m^6^A modification in macrophage metabolic reprogramming. In macrophages stimulated with LPS, oxLDL, or other pro-inflammatory factors, metabolic stress can induce abnormal m^6^A methylation and alter the expression of m^6^A-regulating proteins. Conversely, m^6^A modification can directly impact key enzymes, transporters, and transcription factors in various metabolic pathways, influencing macrophage polarization and the progression of inflammation. However, m^6^A-related studies have predominantly focused on modulating metabolic enzymes indirectly via transcription factors, such as HIF-1α involved in glycolysis. This highlights the need for more research directly targeting specific metabolic enzymes. In particular, studies on how m^6^A regulates the two breakpoints in the TCA cycle are also limited, indicating that several mechanisms remain to be elucidated.

To address these gaps, future research should prioritize uncovering how m^6^A modification directly regulates specific metabolic enzymes in macrophages, including key players in glycolysis, lipid metabolism, OXPHOS, and the TCA cycle. Furthermore, studying the interplay between m^6^A modification and the cellular microenvironment, including factors like hypoxia and nutrient availability, will enhance our understanding of m^6^A’s broader regulatory roles. Finally, developing new m^6^A-targeting small-molecule inhibitors will offer a promising strategy for therapeutic interventions in macrophage-driven inflammatory diseases.
